# Traumatic femoral arteriovenous fistula following gunshot injury: Case report and review of literature

**DOI:** 10.1016/j.amsu.2020.05.016

**Published:** 2020-05-30

**Authors:** Youssef Shaban, Adel Elkbuli, Mark McKenney, Dessy Boneva

**Affiliations:** a Department of Surgery, Division of Trauma and Surgical Critical Care, Kendall Regional Medical Center, Miami, FL, USA; b University of South Florida, Tampa, FL, USA

**Keywords:** Traumatic arteriovenous fistula, Femoral artery injury, Traumatic vascular injury, Complex venous injury, Trauma outcomes, (AVF), arteriovenous fistula, (VIS), venous injury severity, (DVT), deep vein thrombosis, (GSW), gunshot wound

## Abstract

**Introduction:**

Vascular injuries account for approximately 2–4% of trauma admissions with only 2.5% of these being traumatic arteriovenous fistulas (AVFs). We offer a case report of a traumatic AVF and review of the literature.

**Presentation of case:**

A 40-year-old male presented following 4 gunshot wounds, 2 in the forearm and 2 in the left upper thigh. The patient had decreased range of motion and paresthesia of the left lower extremity with palpable pulses and adequate capillary refill in all extremities. A CT angiogram demonstrated a left traumatic AVF involving the left deep femoral artery and left common femoral vein with an adjacent bullet fragment. The patient was taken to the operating room and underwent an exploration of the left groin, repair of the traumatic AVF, and removal of bullet fragment. The venous aspect had a grade IV injury and was ligated. The arterial defect was debrided to healthy tissue and repaired primarily. The patient recovered from his injuries with adequate ambulation and resolution of lower extremity edema. He was discharged home on postoperative day 4 on aspirin and a compression stocking.

**Discussion:**

Traumatic AVFs are rare, with up to 70% diagnosed in a delayed fashion. Clinicians must maintain a high index of suspicion to correctly diagnose and manage this injury to avoid potential morbidity and mortality.

**Conclusion:**

Despite literature accounts of surgeons’ experience, this pathology is lacking level one evidence-based standardized surgical management algorithms. Controversy exists regarding venous repair methods.

## Highlights

• Early diagnosis and management of traumatic AVFs help avoid potential long-term morbidity and mortality.
• Traumatic AVFs with Grade IV venous injuries can be safely ligated with minimal morbidity while reducing operative time and potential blood loss.
• Patients with traumatic AVFs should be placed on a strict DVT surveillance and management regimen along with compression stockings postoperatively.

## 1 Introduction

Vascular injuries account for approximately 2–4% of trauma admissions with only 2.5% of these being traumatic arteriovenous fistulas (AVF). This injury is commonly missed with up to 70% of patients diagnosed in a delayed fashion. Most patients do not present with the hard signs of vascular injury such as loss of distal pulses, expanding hematoma, or pulsatile mass [1]. Potential consequences of an untimely diagnosis include development of a pseudoaneurysm, neuropathy, skin ulceration, thromboembolic sequelae, limb loss, fistula rupture with hemorrhage, and cardiac overload with subsequent cardiac failure or endocarditis [1,2].

Patients may present asymptomatic or with a pulsatile hematoma, bruit, or thrill. A machinery murmur can be auscultated in most patients with a chronic AVF [3]. However, up to 50% of clinical exams have been reported as misleading which explains the difficulty in making the correct diagnosis and the large number of cases being diagnosed in a delayed fashion [2,3].

The gold standard of diagnosis was digital subtraction angiography however this not commonly performed in the presence of a normal neurovascular clinical exam. Less invasive and cost effective tools such as duplex and color Doppler sonography along with CT angiography and MRI have been described. CT angiography has been demonstrated to have sensitivities ranging from 90 to 100% and specificities ranging from 98 to 100% [3].

The pathophysiology of a traumatic arteriovenous fistula incorporates an initial simultaneous injury to an artery and adjacent vein, which subsequently leads to an abnormal communication between the two vessels [3]. The mechanism of injury is frequently penetrating with majority of cases being stab wounds followed by missile or gunshot wounds. In reviewing 210 traumatic arteriovenous fistulas, Robbs et al. observed greater than 50% were located in the cervico-mediastinal vessels followed by 22% in the upper limbs, and 20% in the lower limbs [4]. Over time a missed diagnosis with the continued left-to-right shunt and progressive dilatation of the afferent arteries and efferent veins may lead to heart failure. In addition, venous-lymphatic pathology such as skin ulcerations may occur [1,3]. Clinicians must maintain a high index of suspicion to correctly diagnose and manage this injury in a timely fashion in order to avoid potentially nonreversible morbidity and mortality. This work has been reported in line with the SCARE criteria [5].

### 1.1 Case presentation

**Fig. 1:**
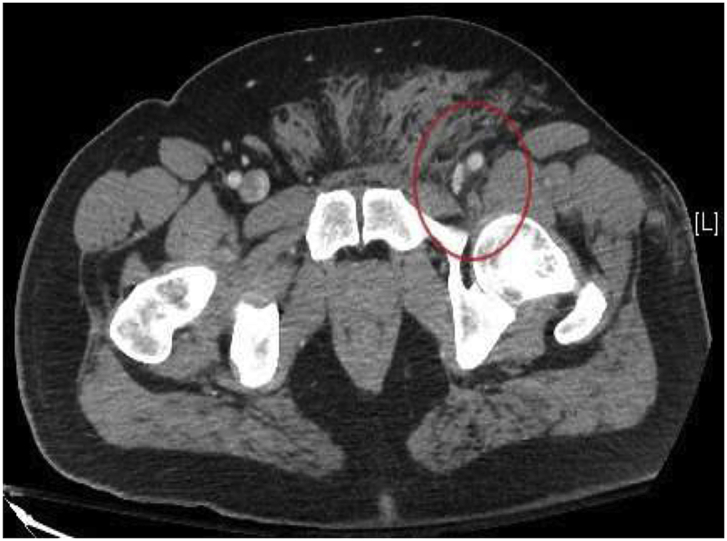
CT axial image of the left common femoral artery and vein just proximal to the traumatic arteriovenous fistula.

A 40-year-old obese male presented to our Level 1 Trauma Center following multiple gunshot wounds (GSWs) to the left upper and lower extremities. The patient was hemodynamically stable and presented with a Glasgow Coma Scale of 15. On exam, there were 4 GSWs with 2 in the left forearm and 2 in the upper left thigh. The patient had decreased range of motion and paresthesia of the left lower extremity but adequate capillary refill in all extremities. There were palpable pulses of the left femoral, popliteal, dorsalis pedis, and posterior tibial arteries. Initial emergency department imaging demonstrated no fractures in the left upper and lower extremities but shrapnel was appreciated. A CT angiogram of the abdomen with runoff demonstrated a left traumatic arteriovenous fistula involving the left deep femoral artery and left common femoral vein with adjacent bullet fragment (Fig. 1, Fig. 2a, Fig. 2b). There was also shrapnel in the soft tissues anterior to the pubic symphysis with associated hematoma and soft tissue inflammation (Fig. 3). Distal vasculature were intact and patent.

**Fig. 2a:**
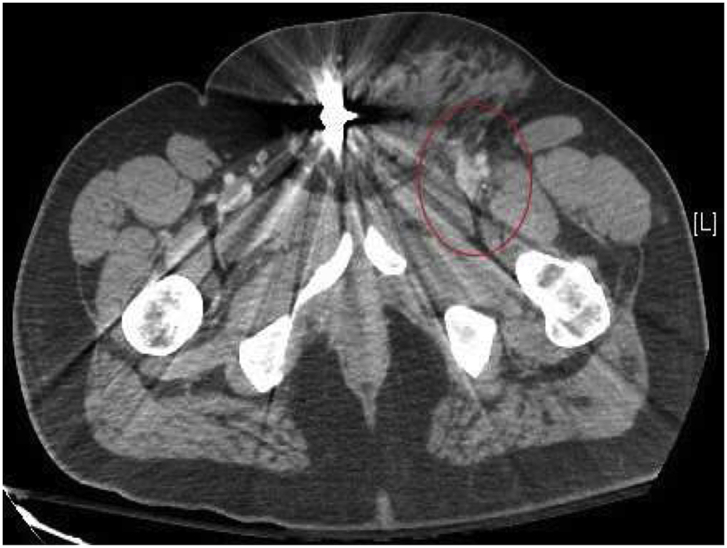
CT axial image of the left traumatic arteriovenous fistula between the deep femoral artery and common femoral vein with the associated perivascular tissue injury.

**Fig. 2b:**
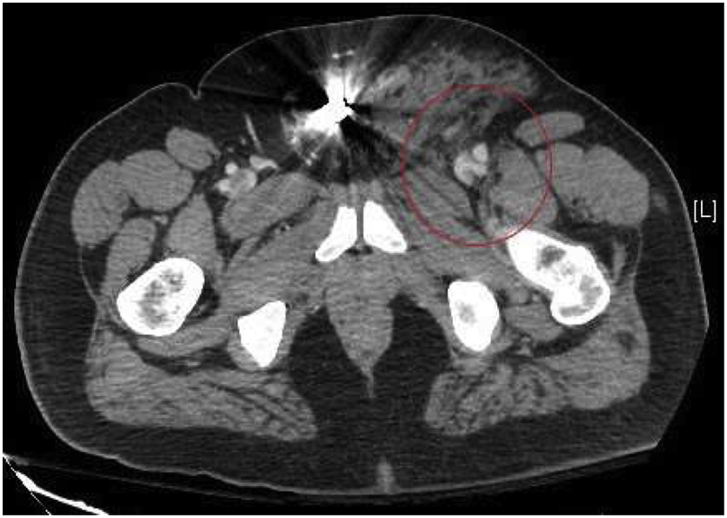
CT axial image of the left traumatic arteriovenous fistula between the deep femoral artery and common femoral vein with the associated perivascular tissue injury.

**Fig. 3:**
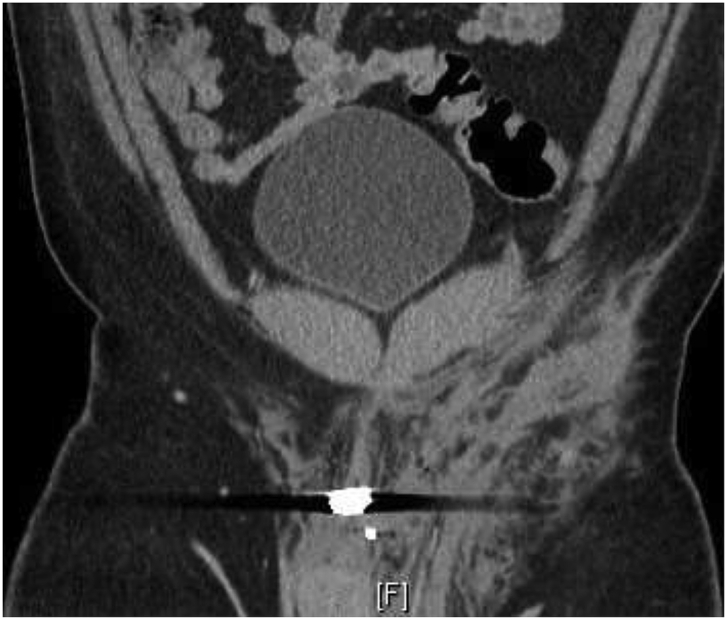
CT coronal image of the abdomen and pelvis illustrating the bullet fragment from the gunshot wound and associated tissue damage and inflammation.

The patient was taken to the operating room and underwent an exploration of the left groin, repair of the traumatic AV fistula, removal of bullet fragment in the suprapubic region. Intraoperatively, proximal and distal control of the deep femoral artery was achieved and the fistula was identified just distal to the take off. The vein had significant perivascular soft tissue injury with greater than 50% venous wall disruption, a grade IV injury. The vein was ligated with combination of sutures and clips. The arterial defect was debrided to healthy tissue and repaired primarily using interrupted 5–0 polypropylene sutures without stenosis (Fig. 4a, Fig. 4b). The left lower extremity was placed in a compression stocking with palpable distal pulses and normal capillary refill.

**Fig. 4a:**
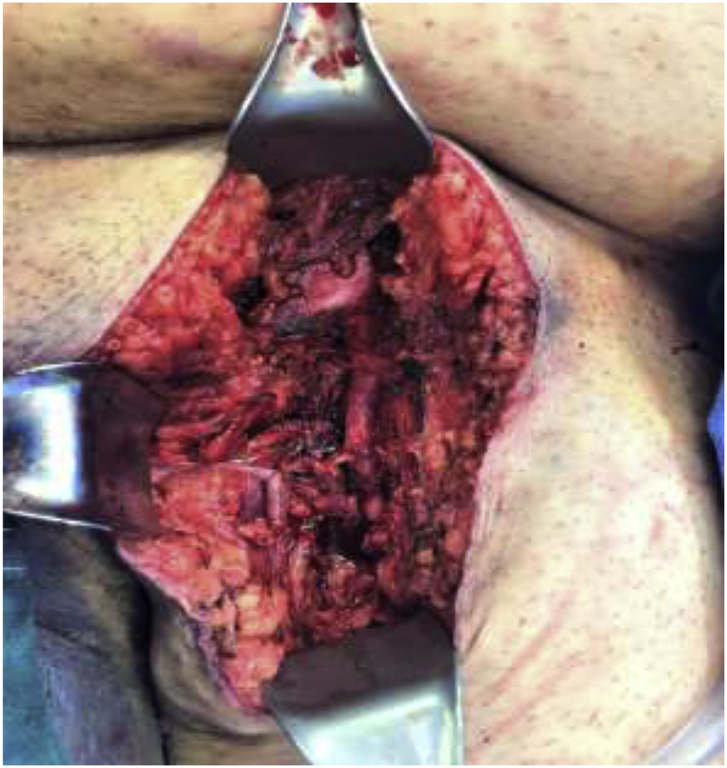
Intraoperative image of the left lower extremity incision on the obese patient. Illustrating the repaired traumatic arteriovenous fistula with the femoral vein ligated. Note the surrounding tissue damage.

**Fig. 4b:**
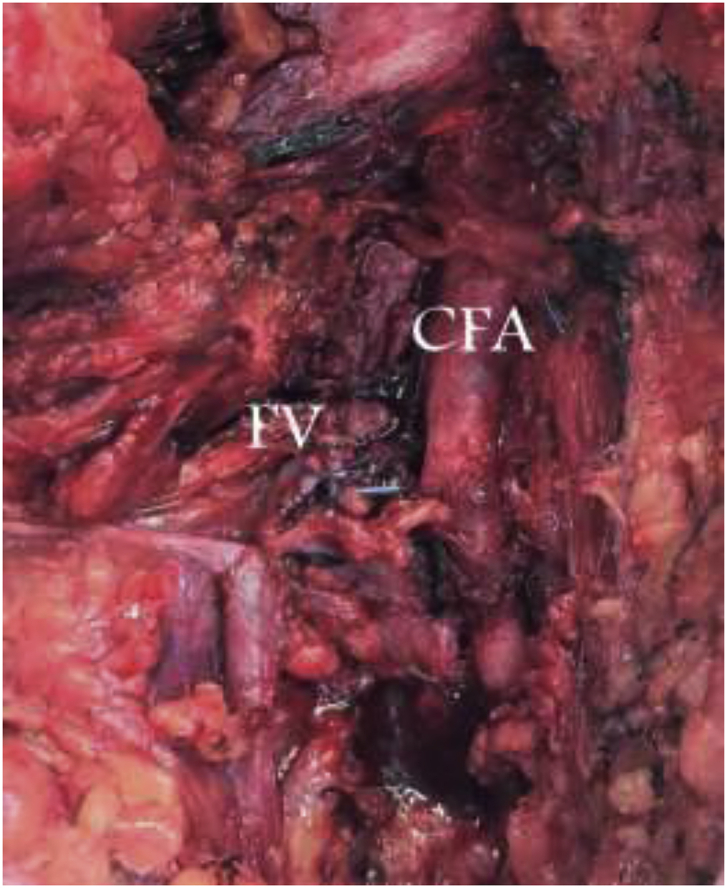
Intraoperative image illustrating the repaired traumatic arteriovenous fistula with the femoral vein ligated. Note the surrounding tissue inflammation. CFA-common femoral artery. FV-femoral vein.

The patient recovered from his injuries with resolution of his paresthesia's and was able to ambulate. He was discharged home on postoperative day 4 on aspirin and a compression stocking. On last follow up 6 months from the initial trauma the patient is asymptomatic and doing well.

## 2 Discussion

Clinicians must maintain a high index of suspicion to correctly diagnose and manage this pathology in order to avoid potential morbidity and mortality. Potential consequences of an untimely diagnosis include development of a pseudoaneurysm, neuropathy, skin ulceration, thromboembolic sequelae, limb loss, fistula rupture with hemorrhagic shock which is potentially lethal, and cardiac overload with subsequent cardiac failure or endocarditis [1,2].

Despite literature accounts of surgeons’ experience with traumatic AVFs, this injury is lacking level one evidence-based standardized surgical management algorithms. This vascular entity happens too infrequently for any single institution to accumulate enough cases for meaningful statistical analysis. Most of the data published are case reports, surgeon experience, and retrospective analysis of hospital specific outcomes.

It is widely agreed upon that early diagnosis and treatment of AVFs is recommended, as this will also prevent subsequent complications [2]. The traditional approach is surgical resection of the fistula with anatomic reconstruction or primary repair, however, other less invasive approaches such as covered stents and coil embolization have been described [2].

In one of the largest studies of traumatic AVFs, Robbs et al. reviewed 202 patients from South Africa, with 210 traumatic AVFs with 98% of injuries resulting from penetrating injuries. The majority of cases were from stab wounds followed by missile wounds at 63% and 26%, respectively. Arterial continuity was established in 80% of cases, mainly by autogenous reconstructions. Venous injuries were treated by ligation or lateral suture. Investigators concluded that patients treated within 1 week of the injury had a lower rate of perioperative mortality and morbidity, mainly due to the subsequent difficulty of controlling vessels following fibrosis and massive venous dilation after one week [4].

Due to the lack of level one evidence-based standardized surgical management algorithms for traumatic AVFs, experts base their treatment on extrapolated data from other vascular injuries. The most controversial subject is venous treatment methods in combined arterial and venous injuries such as in traumatic AVFs [6,7]. Methods include primary repair via lateral suturing or end-to-end anastomosis, ligation, or using an autogenous vein interposition graft or vein patch. Ligation was the standard of care until World War II, however after reviewing cases from the Korean and Vietnam Wars management shifted in favor of primary repair [7]. However the suspected benefits of repairing venous injuries in order to avoid potential sequela of ligation such as chronic venous insufficiency and early postoperative edema has been called to doubt. Critics of complex repairs have shown these complications arising regardless of the type of repair method and suggest the increased complexity and operative time as not warrant a repair [[6], [7], [8], [9]].

In a study comparing venous repair versus ligation, Drs. Yelon and Scalea analyzed 74 patients with 79 venous injuries of the lower extremity or pelvis. The ligation cohort was 48 and 31 for the repair group. Repairs included 2 interposition grafts, 8 end-to-end repairs, 16 venorrhaphies, and 5 vein patches. Authors also introduced a venous injury severity (VIS) system: Grade I: < 50% laceration, Grade II: >50% disruption of venous wall, including AVFs, Grade III: complete venous transection or thrombosis, and Grade IV: >50% wall disruption or venous thrombosis with significant perivascular soft-tissue injury. Patients treated with venous injury had a greater VIS, greater incidence of shock, and higher transfusion requirements. Investigators showed identical morbidity rates with no increased need for fasciotomies in the ligation cohort. Interestingly, as in our case with a Grade IV venous injury, 86% of the ligation cohort were free of edema at discharge. Authors recommend repairing simple venous injuries when feasible in stable patients, however regarding complex venous repairs one must weigh the risks of increased operative time and further blood and heat loss against the possibility of a problem secondary to venous ligation [9].

In a prospective study of 63 patients with either Grade III or IV lower extremity venous injuries treated with ligation, Kurtoglu et al. found no severe chronic venous insufficiency or postthrombotic syndrome with a median follow up of 18 months. In support of Yelon and Scalea, authors propose venous ligation as a safe and effective management alternative for serious Grade III and IV lower extremity venous injuries. Authors propose a strict deep vein thrombosis (DVT) surveillance and management regimen along with compression stockings for patients postoperatively. Perhaps patients may benefit from prophylactic anticoagulation or low dose aspirin postoperatively however more research is needed in this matter.

Interestingly, a recent study by Manley et al. demonstrated patients with venous ligation had fewer episodes of venous thromboembolism (9% vs. 31%, p = 0.02) with no difference in symptomatic lower-extremity edema (37% vs. 39%, p = 0.88) or amputation rates (0% vs. 2%, p = 0.99) [10].

As mentioned previously, the majority of cases are commonly missed and up to 70% of patients are diagnosed in a delayed fashion [1]. In a rare observation at the natural history of traumatic AVF over 50 years following the initial injury, Chaudry et al. describe a case of gentlemen who was incidentally diagnosed with a traumatic AVF of the right profunda femoris artery and vein presenting with aneurysmal ileo-femoral arteries and veins and pulmonary hypertension. Authors successfully performed a hybrid open and endovascular approach with an Amplatzer II plug deployed beyond the AVF in the distal right deep femoral artery, and an 18-mm-diameter Amplatzer II plug deployed within the AVF. Follow up CT angiography demonstrated an excluded AVF without flow. The iliac venous system had returned to normal diameter [11].

Our patient suffered a left femoral AVF following multiple GSWs that was successfully diagnosed and treated in a timely manner. Due to the extent of the venous injury of the AVF we opted for ligation and provide a satisfactory outcome. Although the authors agree with the general approach of repairing the vein when feasible we provide an adequate alternative that reduces potential operative time and subsequent blood loss. Aggressive postoperative physical therapy and continued use of compression stocks allowed for his lower extremity edema to resolve prior to discharge. The patient did suffer a DVT complication, however, he admittedly was non-compliant with the compression stocking and this highlights the importance of a strict DVT surveillance and management protocol postoperatively.

## 3 Conclusion

Traumatic arteriovenous fistulas are a rare entity that is commonly missed with up to 70% of patients diagnosed in a delayed fashion. Clinicians must maintain a high index of suspicion to correctly diagnose and manage this pathology in order to avoid potential morbidity and mortality. Despite literature accounts of surgeons’ experience, this pathology is lacking level one evidence-based standardized surgical management algorithms. Controversy exists regarding venous repair methods in traumatic AVFs. We present an interesting case of a traumatic femoral arteriovenous fistula with a Grade IV venous injury following multiple gunshot injuries that was successfully treated with venous ligation and primary repair of the artery.

## Ethical Approval

This case report was conducted in compliance with ethical standards. Informed written consent has been obtained and all identifying information is omitted.

## Sources of funding

None.

## Author contribution

YS, AE, DB, MM– Conception of study, acquisition of data, analysis and interpretation of data.

DB, MM – Management of case YS, AE, DB, MM –drafting of abstract, drafting of manuscript, critical revision of manuscript.

AE, YS, DB, MM – Approval of the final version for submission.

## Trial registry number

NA.

## Research Registration Unique Identifying Number (UIN)

This is a case report study.

## Guarantor

Dessy Boneva.

Mark McKenney.

## Provenance and peer review

Not commissioned, externally peer reviewed.

## Declaration of competing interest

None.
